# Improvement in the Damping Behavior of Hierarchical Carbon Fiber-Reinforced Plastic for Park Golf Club Faces

**DOI:** 10.3390/polym17030264

**Published:** 2025-01-21

**Authors:** Seoyeon Bae, Minhyeok Shin, Eunjung Kim, Sungbi Lee, Woong-Ryeol Yu, Cheol-Hee Ahn, Wonjin Na

**Affiliations:** 1 Composite Materials Application Research Center, Korea Institute of Science and Technology (KIST), Wanju-gun 55324, Republic of Korea; bbbbseo@kist.re.kr (S.B.); t24088@kist.re.kr (M.S.); i032132@naver.com (E.K.); 2 Department of Materials Science and Engineering, Research Institute of Advanced Materials (RIAM), Seoul National University, 1 Gwanak-ro, Gwanak-gu, Seoul 08826, Republic of Korea; leesungbi@kist.re.kr (S.L.); woongryu@snu.ac.kr (W.-R.Y.); chahn@snu.ac.kr (C.-H.A.); 3 Department of JBNU-KIST Industry-Academia Convergence Research, Jeonbuk National University, Deokjin-gu, Jeonju-si 54896, Republic of Korea

**Keywords:** carbon fiber composite, park golf club, nanofillers, coefficient of restitution, vibration damping

## Abstract

Park golf, introduced to Korea in 2000, has become a popular leisure activity, especially among older people. However, sudden shock between the ball and carbon fiber-reinforced plastic (CFRP) face can increase the risk of injuries, highlighting the need for enhanced damping material. However, restitution and damping are critical properties of golf clubs and often exhibit a conflicting relationship; thus, a method is needed to address this challenge. Therefore, this study aimed to develop a CFRP with an enhanced restitution and damping ratio by incorporating carbon nanotubes and graphene oxide nanofillers into the existing CFRP face material. A drop test apparatus was set up to measure the coefficient of restitution, and the damping properties were evaluated using a pencil lead-breaking test. CNTs exhibited high rebound properties due to their stiffness and hardness. In contrast, GO provided a modest increase in rebound while demonstrating a superior damping ratio, attributed to its layered structure and high internal friction. Based on these results, the optimal nanofiller content was determined as GO 0.025 wt%, showing a minor improvement in rebound performance, a 1033% improvement in vibration damping, and an 84% improvement in acoustic damping. Notably, this finding implies the importance of nanomaterial shape and its interaction with the composite matrix. A double-masked user test with a prototype confirmed enhanced comfort and reduced vibration feedback. The low-vibration components developed in this study are expected to be applicable in future research for controlling the damping ratio under impact or vibrations, such as UAM and helicopters.

## 1. Introduction

Park golf originated in Japan in 1983 and was introduced to South Korea in 2000, becoming a popular leisure activity [1]. The golf players (called ‘parkers’) use a club with a wooden clubhead and a carbon fiber-reinforced composite (CFRP) impact face. It is trendy among the older generation due to its high accessibility, minimal preparation, and shorter playing time. However, unexpected movements during swings can lead to tendon injuries, such as epicondylitis [2]. Although there are no precise statistics on injury cases related to park golf, the number is expected to increase with the growth of its market, similar to trends in other types of golf [3,4,5]. Efforts to prevent injuries have included adding cushioning behind the striking surface. High-modulus polymeric fibers have also been incorporated into the club face to enhance shock absorption. However, further shock absorption during the impact is still required for the safety of players, as current solutions have shown limitations in completely mitigating impact forces, leading to a risk of injuries such as tendonitis and joint strain.

The current CFRP face material used in park golf clubs offers high stiffness and is lightweight, but it also has limitations regarding shock absorption and vibration damping. Several approaches to enhance vibration damping have been developed. One fundamental approach involved using a thermoplastic matrix or another high-damping fiber system, such as natural fibers [6,7]. Differences in fiber structures, such as ply stacking sequences and ply thickness [8,9], have also been analyzed. Furthermore, the incorporation of nanofillers is an effective strategy considering the minimal structural changes required and their mechanical properties. Previous studies have shown enhancements in the vibration-damping properties of polymer nanocomposites [10,11]. Pan et al. [12,13] showed vibration damping using carbon nanotubes (CNTs) and graphene nanoplatelets (GNPs), with 40% and 128% increases in the first-order loss factors in both nanofillers. Gong et al. [14] investigated the damping properties of CFRPs containing graphene oxides (GOs), reporting that the damping loss factor of CFRPs can increase by 113%. Kim et al. [15] also investigated a hierarchical CNT-containing CFRP structure, showing a 400% increase in the loss factor. Feng et al. [16] demonstrated a vibration-damping sandwich structure using functionalized reduced graphene oxide (rGO), combining the material and structural approaches. Chandramohan et al. [17] investigated a synergistic effect of nanofillers using graphene and carbon black. In summary, most nanocomposites can contribute to improved damping properties and two-dimensional materials usually show higher damping properties due to higher interfacial damping. However, to the authors’ knowledge, rebound and damping properties have not been correlated in an actual application, which motivated this research. For park golf clubs, it is crucial to maintain rebound performance while achieving vibration damping, making it essential to investigate the optimal combination. This would also lead to other applications relevant to impact scenarios [18,19,20,21].

The aim of this study was to design a hierarchical CFRP material with one- and two-dimensional nanofillers, CNTs, and GOs, and evaluate the restitution performance and damping properties. The possibility of enhancing these two critical properties was investigated, addressing the longstanding trade-off between impact resistance and vibration damping in conventional composites. An apparatus evaluating the rebound properties was designed, and the coefficient of restitution was measured. The pencil lead-breaking (PLB) test was used to evaluate the damping property. The contents of nanofillers were optimized. An industrial-scale prepreg material with optimized GO contents was manufactured, and the mechanical properties were measured. Finally, park golf club prototypes were produced and tested by users to identify actual comfort during impact.

## 2. Materials and Methods

### 2.1. Material and Specimen Preparation

Carbon fiber (CF) fabrics produced from Toray T-300 carbon fiber tows (C-120, 200 g/m^2^ areal weight; Minhu Composite, 32 Science Industrial Complex 1-ro 60beon-gil, Gangseo-gu, Busan 46742, Republic of Korea) were used as reinforcement. Epoxy resin (YD-115; Kukdo Chemical, 61 Gasan Digital 2-ro, Geumcheon-gu, Seoul 08588, Republic of Korea) and a curing agent (Jeffamine D-230; Huntsman, The Woodlands, 10003 Woodloch Forest Drive, TX 77380, USA) were prepared as a matrix. For the nanofillers, CNTs (Jenotube 10B; JEIO, 263 Central-ro, Yeonsu-gu, Incheon 22006, Republic of Korea) and GOs were prepared using Hummer’s method (HGO6D30-FM, Powder; GrapheneAll, Yeongcheon-dong, Hwaseong, Republic of Korea).

**Figure 1:**
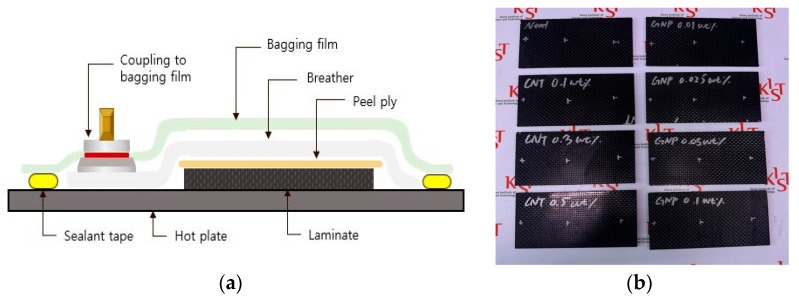
(**a**) Schematic diagram of vacuum bag molding. (**b**) Fabricated specimens.

Neat (without nanofillers) and hierarchical specimens were fabricated. CNT content varied from 0.1 to 0.5 wt%, and GO content varied from 0.01 to 0.1 wt%. The nanofillers were mixed with epoxy resin using a three-roll mill (E80; EXAKT Technologies, Heidbergstraße 70, 22851 Norderstedt, Germany) under 200 rpm with controlling gaps. The gaps were 75/25 µm for two passes, 45/15 µm for one pass, and 30/10 µm for one pass. Composite specimens were fabricated via hand layup impregnation and vacuum bag molding. Eight plies of CF fabrics were stacked, and vacuum bag molding was set up on a hot plate, as shown in Figure 1a. The prepared materials were layered in the following order: release film, breather, and bagging film, with sealant tape reinforcing the bagging film to maintain the vacuum. A vacuum pump maintained below 13 Pa (0.1 Torr) was used, ensuring a 1 atm pressure difference. The forming was performed at 70 °C for 4 h. The specimens were produced with dimensions of 110 × 60 × 1.8 mm^3^. The fabricated laminates had a fiber fraction of 48 vol% and a void content of around 7 vol%. A total of 8 laminate cases were prepared (Figure 1b and Table 1) for rebound, PLB damping, and dynamic mechanical analyzer (DMA) tests. Those laminates were processed into specimen geometry with a waterjet cutting machine (Superjet-T500; TOPS, 54 Ansan-gil, Danwon-gu, Ansan 15420, Republic of Korea). The dispersion state of nanofillers within the epoxy matrix was analyzed using microscope analysis. A transmission electron microscope (TEM, Titan G2 Cubed 60-300; Thermo Fisher Scientific, 168 3rd Ave, Waltham, MA 02451, USA) was employed. TEM images were acquired at an accelerating voltage of 80 kV with an Image-Cs corrector.

The CF prepreg was fabricated based on the optimal composition (Section 3), which showed the best vibration-damping property. Unidirectional (UD) and plain-woven prepregs with nanofillers were manufactured. Four types of prepregs and subsequent specimens (neat UD, neat woven, nanofiller containing UD, and nanofiller containing woven fabric) were prepared with the same Toray T300 carbon fibers and fabrics. The prepregs were obtained from a specialized manufacturing company (KGF, 101 Wonmanseong-ro, Deokjin-gu, Jeonju, 54853, Republic of Korea), and the resin was prepared with epoxy resin (YD-128; Kukdo Chemical, Republic of Korea), a hardener (dicyandiamide, DICY; Kukdo Chemical, Republic of Korea), and a curing accelerator (3-(3,4-chlorophenyl)-1,1-dimethylurea, DCMU; Kukdo Chemical, Republic of Korea). The resin contents were controlled at 33%. The final thickness of the woven prepreg ply was 0.3 mm, and that of the UD prepreg ply was 0.23 mm. The fabricated prepregs were cut into dimensions of 400 × 400 mm, and an autoclave process was performed at 0.7 MPa (7 bar) pressure, with curing at 80 °C for 30 min and 125 °C for 2 h. The laminates were used to manufacture the prototype clubs.

### 2.2. Measurement of Coefficient of Restitution (COR)

**Figure 2:**
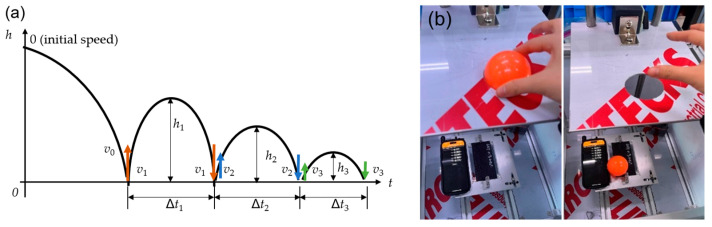
(**a**) Schematic diagram of determining the coefficient of restitution; (**b**) the apparatus for the rebound test.

The coefficient of restitution (COR, *e*) was measured by calculating the height and velocity based on the impact of a falling ball on a fixed CFRP plate. The ASTM F1887 standard in [22] was referred to for the design of the testing apparatus and methodology. The COR is defined as the ratio of the separation velocity after a collision (*v*_2_) to the velocity of the approach before the collision (*v*_1_) [23,24,25]. When the ball impacts the plate for the first time (denoting the velocity before the first impact as *v***_0_**), it repulses with the initial velocity *v*_1_ up to height *h*_1_ for the time interval Δ*t*_1_/2 (Figure 2a). The velocity decreases due to gravity, so the relationship between *v*_1_ and Δ*t*_1_ is expressed in Equation (1). The relationship is the same for *v*_2_ and Δ*t*_2_.

(1) $$v_{1}=g\left(\frac{{∆t}_{1}}{2}\right)$$

Consequently, the first-order COR can be expressed by Equation (2). The second- and third-order CORs are the same.

(2) $$e_{1}=\frac{{∆t}_{2}}{{∆t}_{1}}$$

A drop test apparatus was designed to implement the measurement, featuring an open central area and adjustable fixation points at two or more corners to hold the specimen (Figure 2b). The sample was fixed by tightening bolts at the bottom plate, and the experiment involved dropping a park golf ball (60 mm diameter, 95 g weight, HIMILAN^TM^ ionomer resin shell and filled with polyurethane foam) onto the specimen from a 260 mm height. The time intervals Δ*t*_1_, Δ*t*_2_, Δ*t*_3_, and Δ*t*_4_ were measured using the acoustic sequential stopwatch of the Phyphox^®^ app in the Android 13 environment in a smartphone (SM-G996N; Samsung Electronics, 129 Samsung-ro, Yeongtong-gu, Suwon 16677, Republic of Korea). The coefficients of restitution, *e*_1_, *e*_2_, and *e*_3_, were calculated and averaged. Ten tests were performed, and five data points were selected for each specimen.

### 2.3. Vibration Damping

The vibration damping was evaluated via two methods: DMA and PLB tests. The DMA analysis was performed at room temperature (298 K) using a dynamic mechanical analyzer (DMA Q800; TA Instruments, 159 Lukens Drive, New Castle, DE 19720, USA). Specimens were prepared at 13 × 35 × 1.8 mm^3^. Four specimens were tested in each material. They were clamped at one end using a stationary clamp, and a flexural strain of 0.1%/min was applied at the other end using a movable clamp (single-cantilever mode). A frequency sweep from 1 Hz to 100 Hz was conducted at a fixed room temperature. The loss factor (tan*δ*) was measured to evaluate the vibration-damping properties.

**Figure 3:**
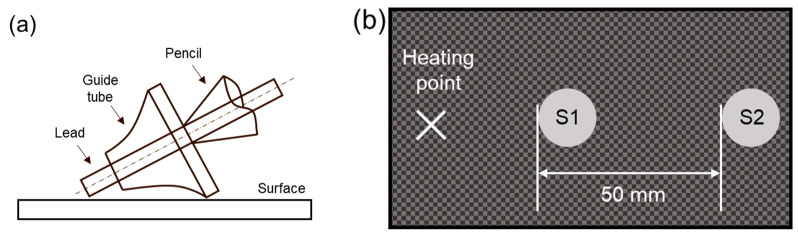
(**a**) Specially designed pencil for the PLB test. (**b**) Schematic diagram of heating point and sensor location.

To evaluate the acoustic damping property and vibration-damping performance, especially at high frequencies, pencil lead-breaking (PLB) tests were performed following ASTM E976 [26]. The PLB test assessed the wave propagation and amplitude dissipation of a lamb wave. The Hsu–Nielsen source (H-N source) and two acoustic emission (AE) sensors (IDK-AES-H150; IDK Co., Ltd., 35 Techno 9-ro, Yuseong-gu, Daejeon 34027, Republic of Korea) were prepared (labeled as S1 and S2). A specially designed H-N source incorporating a lead guide tube, following ASTM E976, was prepared to impact the sample (Figure 3a). Two AE sensors were attached to each specimen using silicone grease (HIVAC-G; Shin-Etsu, 2-6-1 Otemachi, Chiyoda-ku, Tokyo, Japan). The AE signals were amplified using pre-amplifiers (IDK-AMP-S001-40/60; IDK Co., Ltd., Republic of Korea) with a gain of 40 dB_AE_ (i.e., 100 times in voltage). AE signals over a 40 dB_AE_ threshold were recorded during the test using a data acquisition system (IDK-DAQ8; IDK Co. Ltd., Republic of Korea) with a 5 MSPS sampling rate. The peak definition time (PDT) was set to 200 µs, the hit definition time (HDT) was set to 400 µs, and the hit lockout time (HLT) was set to 4000 µs. The relative sensitivity between S1 and S2 was calibrated before the PLB test. S1 was located at a proximal distance (60 mm from the source), and S2 was located at a distal distance (110 mm from the source). The two sensors had a 50 mm propagation length (Figure 3b). The 4H pencil lead was fractured by hand after contact. The vibration-damping ratio (*δ*_PLB_) was calculated using amplitudes *A*_1_ and *A*_2_ and distance following Equation (3). Twenty tests were performed for each specimen.

(3) $$\delta_{PLB}=\frac{A_{1}-A_{2}}{50}(dB/mm)$$

### 2.4. Mechanical Testing and User-Based Performance Evaluation

Tensile tests followed the ASTM D3039 standard [27]. Specimens were prepared at 20 × 180 × 1.8 mm^3^. Glass fiber-reinforced plate taps were attached to 40 mm of each end, aiming for a 100 mm gauge length. Tensile tests were conducted using a universal testing machine with a load cell of 250 kN (Instron 5985; Instron, 825 University Avenue, Norwood, MA 02062, USA) and a crosshead speed of 0.5 mm/min.

A prototype park golf club with an 8 mm-thick face was manufactured using the prepregs in Section 2.1, and a real-user evaluation was conducted. Woven fabrics were located at the top and bottom, and the internal structure was designed as a cross-ply structure. Ahwagolf (1009-1 Dongseo-daero, Seo-gu, Daejeon 35289, Republic of Korea) processed the park golf club face to produce the prototype clubs. The same design as the existing Ahwagolf product line was used, along with an identical ash wood clubhead. A neat park golf face and a park golf face prototype were manufactured. A double-masked swing test was conducted. Ten amateur parkers with average skills were recruited. The users, who were unaware of whether the park golf club contained nanofiller, performed ten swings. The users evaluated the impact sound, impact feeling (qualitative smooth feeling during impact), flying distance, and vibration damping on a scale from 1 to 5.

## 3. Results and Discussion

### 3.1. Morphological Analysis of Nanofiller Dispersion in CFRPs

**Figure 4:**
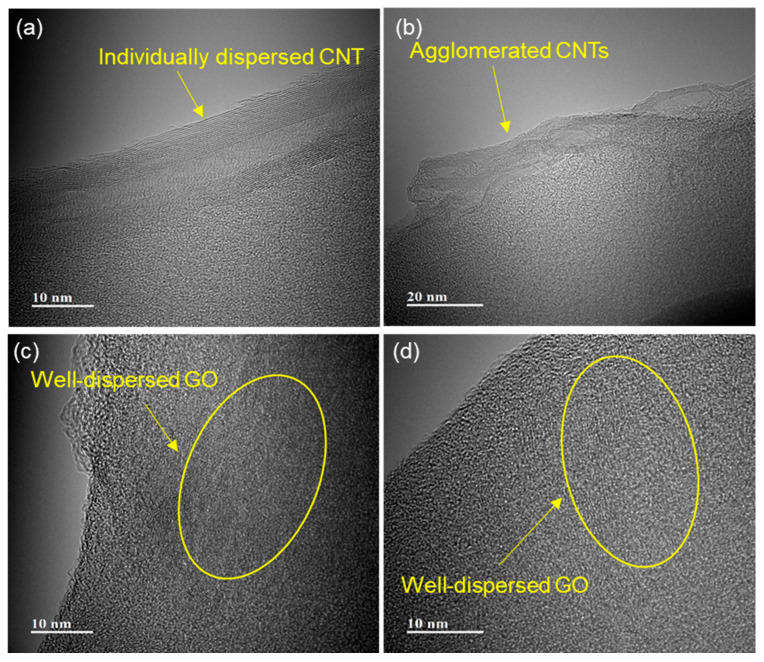
TEM images of (**a**) C01, (**b**) C05, (**c**) G025, and (**d**) G100.

Figure 4 shows the dispersion states of CNT and GO nanofillers in CFRPs. For the C01 sample, no agglomeration was observed and the CNTs were well separated, indicating a uniform dispersion. In contrast, the C05 sample (higher concentration) displayed predominantly well-dispersed CNTs, although minor agglomeration was observed in certain localized regions. In the case of GO nanofillers, both the G025 and G100 samples exhibited excellent dispersion, with minimal signs of agglomeration. The superior dispersion observed in GO samples can be attributed to the low filler concentration and the two-dimensional nature of GO, which facilitates the exfoliation of graphitic layers under the applied shear force during the mixing process. However, in the G100 sample, a small number of multilayered structures (indicative of slight agglomeration) was identified. These findings highlight the influence of filler concentration and morphology on the dispersion quality, with GO demonstrating a notable advantage due to its structural characteristics.

### 3.2. Rebound Test (Coefficient of Restitution)

**Figure 5:**
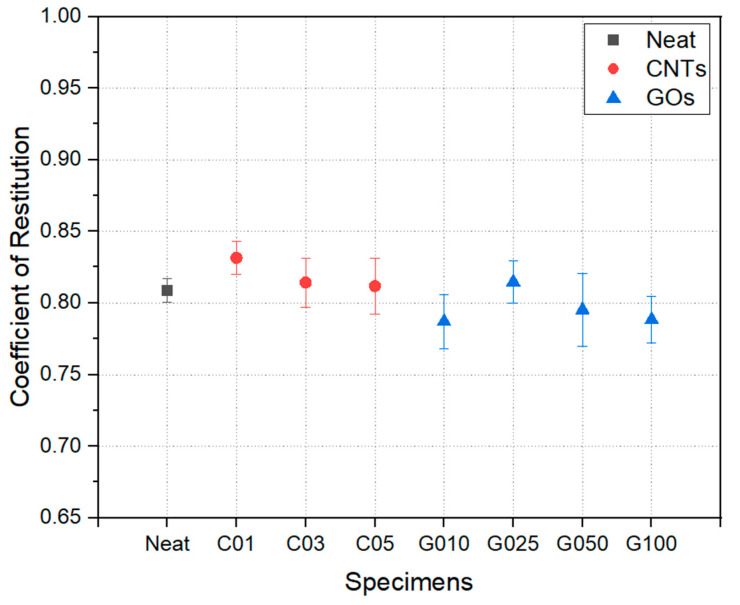
Coefficient of restitution of CFRP laminates.

The COR results are shown in Figure 5. The neat specimen recorded an average COR of 0.809, close to the upper limit set by golf club regulations (noting that the coefficient should be below 0.83 when a golf ball strikes a rigid plate or dense wood [28]). The COR increased when CNT was added as a nanofiller. Specifically, the sample with 0.1 wt% CNT (C01) showed the best result, with a coefficient of 0.832. Although adding CNT above 0.1 wt% could improve COR, the improvement was less than that of the C01 case. Therefore, it can be concluded that 0.1 wt% of CNT is the optimal concentration for high-rebound applications.

This increase in coefficient is interpreted as a reinforcement of the epoxy resin, which is directly impacted by the surface of the park golf ball. According to the classical Hertz model [29,30], the collision of an elastic body is a function of the contact point’s stiffness coefficient, radius, and penetration depth. A slight increase in the stiffness of the resin part may not significantly contribute to the overall stiffness coefficient of the composite material. Still, it can locally lead to a reduction in the penetration depth during impact. After impregnation, a resin-rich region exists on the surface of the composite materials and directly impacts interactions with external bodies. The increased modulus may contribute to a reduction in penetration depth. After the CNT content exceeds 0.1 wt%, the increase in COR becomes less significant. The diminishing improvement in COR with CNT contents above 0.1 wt% can be attributed to the agglomeration of nanofillers, which reduces their dispersion efficiency and limits their reinforcement effect. This behavior aligns with prior studies indicating that nanofiller dispersion significantly impacts material properties.

The rebound performance was not improved when GO was used as a nanofiller, except in the case of G025. In this case, adding GO is also expected to slightly enhance the epoxy resin’s stiffness, and the contribution to the reduction in penetration depth is understood to be less significant for GO. The relatively modest effect of GO on COR compared to CNT is likely due to its two-dimensional structure, which interacts differently with the resin matrix. The surface interaction of GO may be less effective in locally increasing the stiffness of the resin-rich region during impact. According to the Hertz model, the observed increase in COR is primarily attributed to the localized reduction in penetration depth, which results from the enhanced stiffness of the resin matrix due to nanofiller incorporation. Consequently, the overall high coefficient of restitution can be attributed to the increased rigidity of the resin due to the incorporation of nanofillers.

### 3.3. Enhanced Vibration Damping in CFRP: Effects of CNT and GO Nanofillers

**Figure 6:**
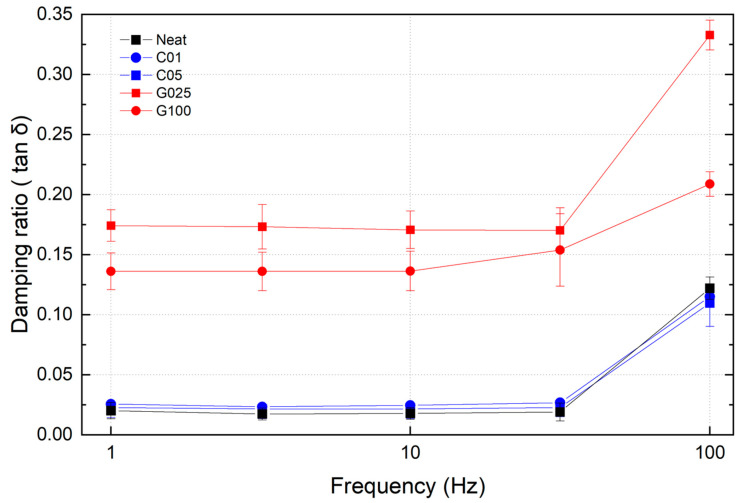
The damping ratio of CFRPs with CNT and GO nanofillers.

The DMA results for the damping ratios (tan δ) across the frequency range of 1–100 Hz are shown in Figure 6. In most cases, the damping ratio remained nearly constant up to 30 Hz and then significantly increased at 100 Hz, with the sole exception being the G100 sample, which showed a clear increase at 30 Hz. The neat specimen exhibited damping ratios between 0.015 and 0.020 up to 30 Hz, rising to 0.122 at 100 Hz. For CNT-containing samples (C01 and C05), the damping ratios showed a marginal increase compared to the neat specimen, remaining nearly identical to each other. In contrast, GO-containing specimens demonstrated a substantial enhancement in damping performance. The G025 sample exhibited a damping ratio of 0.170–0.175, reflecting a remarkable 1033% increase compared to the neat specimen. Its maximum damping ratio reached an impressive 0.333 at 100 Hz, significantly surpassing all other samples. While the G100 sample showed slightly lower damping properties than G025, it still outperformed both the neat and CNT-reinforced specimens. These results underscore the superior damping effect of incorporating GO, a two-dimensional material, into the CFRP matrix. Notably, even with minimal filler content, such as in G025, the damping ratio increased by over 1000%, highlighting the transformative potential of GO in enhancing vibrational energy dissipation in composite materials.

**Figure 7:**
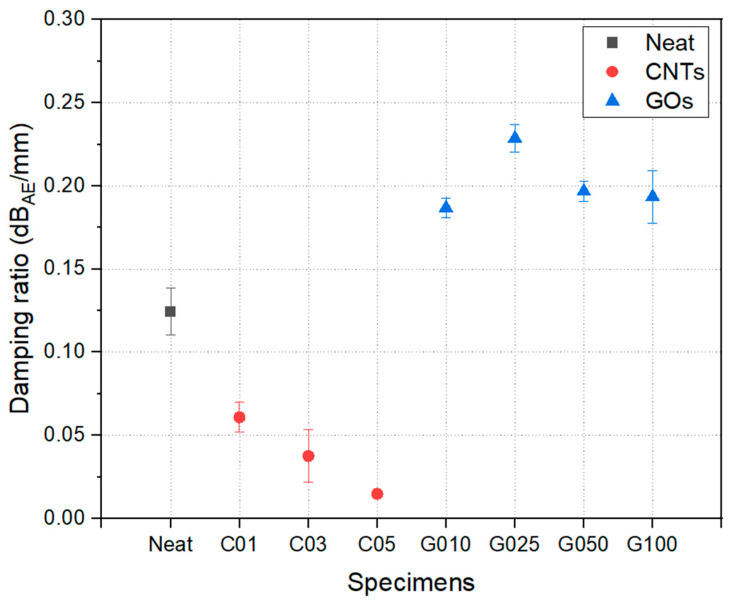
Vibration-damping ratio of CFRP laminates.

The PLB test results are shown in Figure 7. The vibration-damping ratio of the neat specimen was 0.1243 dB/mm. It decreased by more than 0.1 dB/mm when CNT was added, indicating reduced attenuation of acoustic vibration. This suggests that the reinforcement with CNT resulted in less damping of elastic waves, leading to sharper high-frequency impact sounds and increased vibrations in the park golf clubs. In contrast, the highest value of 0.2287 dB/mm for the GO nanofiller was recorded at 0.025 wt%, representing a maximum increase of 84%. This demonstrates that the addition of GO significantly enhanced the vibration-damping effect. In addition, the optimal composition of GO was observed at 0.025 wt%, beyond which, it showed a negative effect. This is likely due to excessive addition leading to nanofiller aggregation, which hinders interfacial slip and reduces vibration-damping efficiency [31]. 

As noted in previous literature, the primary mechanism of vibration damping is the energy dissipation effect due to micro-slip at the interfaces (the slip–stick effect) [32]. In the case of CNT addition, this effect was not prominent, and the improvement in surface wave transmission efficiency due to increased stiffness was more significant. The reduced damping efficiency observed with CNT addition can be attributed to its high stiffness, which enhances the transmission of surface waves but limits the energy dissipation typically caused by interfacial micro-slip. On the other hand, adding 2D materials such as GO effectively enhanced the slip–stick effect on the surface. For GO, the optimal damping performance at 0.025 wt% is likely due to its uniform dispersion and effective enhancement of the slip–stick mechanism. Beyond this concentration, aggregation reduces interfacial slip and damping efficiency.

While rebound distance is essential in park golf, user comfort related to vibration damping and reduced impact noise (often described as a ’smooth swing’) is equally critical. The enhanced damping effect of G025 translates directly to improved user comfort, as evidenced by smoother swings and reduced impact noise. These features are critical for park golf clubs, where performance and user experience are paramount. The lower vibration damping observed with CNT addition is a disadvantage in park golf clubs. The optimal material combination for park golf clubs is G025, which showed significantly improved vibration damping without compromising rebound performance.

### 3.4. Mechanical Property and User-Based Performance Evaluation

Tensile testing was conducted on the prepreg-based product containing 0.025 wt% GO, and the results are presented in Table 2. The UD composite exhibited linear mechanical behavior during testing. The tensile strength of the GO-added sample was 1173 MPa, representing a 7.6% increase compared to 1090 MPa for the sample without GO. The modulus values were of a similar level, measuring 128.0 GPa and 124.4 GPa, respectively. The elongation also remained at a comparable level, with a slight increase. Elongation is not a critical factor for park golf clubs operating primarily within the elastic range. However, since repetitive impacts may partially damage the fibers over time, it is essential to maintain elongation at least at the same level as the conventional product. In this regard, the developed product demonstrates improved safety and reliability compared to the existing materials.

**Figure 8:**
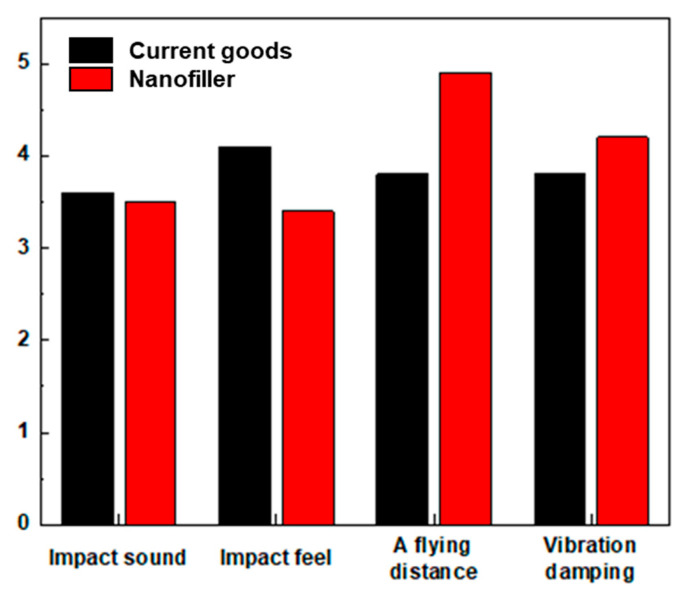
Vibration-damping rate measurement results by material.

The user-based performance testing results showed an advancement in the park golf club swinging experience for users (Figure 8). The current golf club scored an average of 3.65, and the golf club with nanofillers (0.025 wt% GO) scored an average of 4.18. Previous studies have primarily relied on conventional testing methods, such as DMA and tensile testing, which evaluate static or quasi-static properties. While these methods provide valuable insights, they often fall short of capturing the dynamic behavior of materials under real-world conditions. In this study, rebound tests and PLB tests were employed to evaluate restitution and damping properties under dynamic conditions that closely simulate the actual usage environment of park golf clubs. This novel approach facilitated the optimization of nanomaterial content for park golf clubs and contributed to the development of testing methodologies that align more closely with industrial demands. Furthermore, this research bridges the gap between laboratory findings and practical applications by demonstrating industrial-scale fabrication of prepregs and validating performance through user-tested prototypes. The results underscore the potential of these materials for broader applications in low-vibration environments, such as helicopter blades and rotating machinery, where simultaneous improvements in restitution and damping are critical.

## 4. Conclusions

This study focused on the development of a park golf club face composed of carbon fiber-reinforced polymer (designed to impact a plastic ball directly). In the development, the rebound performance and vibration-damping properties mainly focused on user safety. Nanofillers were introduced to achieve enhanced damping without compromising the mechanical properties or altering the structure of existing park golf club faces. This study comprehensively investigated the incorporation of CNT and GO nanomaterials into CFRP face materials for enhanced restitution and damping properties, in addition to the influence of the nanomaterial shape. One-dimensional carbon nanotubes (CNTs) and two-dimensional graphene oxides (GOs) were incorporated. The rebound properties (COR) and vibration damping were quantitatively measured. The results showed that CNT addition led to a high COR of up to 0.832, while GO addition provided enhanced vibration-damping effects of up to 0.2156 dB_AE_/mm. Notably, a small addition of 0.025 wt% GO resulted in an over 1033% improvement in vibration and acoustic damping. Furthermore, positive results were obtained from actual product evaluations, confirming industrial feasibility. Contrary to the conventional expectation that nanomaterials consistently improve damping properties through slip–stick mechanisms, our findings reveal that damping performance may decrease under certain conditions, particularly in acoustic wave propagation. This behavior highlights the importance of the nanomaterial shape and its interaction with the composite matrix. These results address the longstanding dilemma of balancing impact resistance and vibration damping and offer new insights into the design of nanomaterial-reinforced composites for broader applications. This indicates that the developed material could benefit future applications where vibration damping is crucial, such as in helicopters or urban air mobility (UAM) systems continuously exposed to low-level vibrations.

## 5. Patents

The data from this study are published under Korean Patent no. 10-2024-0091675 (Title: COMPOSITE MATERIAL FOR FACE OF PARK GOLF CLUB; Filing Date: 11 July 2024).

## Figures and Tables

**Table 1:** Sample name and material composition.

Sample Name	Filler Content. wt%	Sample Name	Filler Content. wt%
Neat	-	G010	GO 0.01
C01	CNT 0.1	G025	GO 0.025
C03	CNT 0.3	G050	GO 0.05
C05	CNT 0.5	G100	GO 0.1

**Table 2:** Mechanical properties of UD CFRPs with and without GO nanofillers.

Specimens (Unit)	Young’s Modulus (GPa)	Tensile Strength (MPa)	Elongation (%)
Neat UD	128.0	1090	0.905
GO-added UD	124.4	1173	0.913

## Data Availability

The original contributions presented in this study are included in the article. Further inquiries can be directed to the corresponding author.
